# Genome-Wide Identification and Comparative Analysis of MYB Transcription Factor Family in *Musa acuminata* and *Musa balbisiana*

**DOI:** 10.3390/plants9040413

**Published:** 2020-03-27

**Authors:** Lin Tan, Usman Ijaz, Haron Salih, Zhihao Cheng, Nwe Ni Win Htet, Yu Ge, Farrukh Azeem

**Affiliations:** 1 Haikou Experimental Station, Chinese Academy of Tropical Agricultural Sciences (CATAS)-Hainan Key Laboratory of Banana Genetic Improvement, Haikou 571101, Hainan, China; tanlin@catas.cn (L.T.); salih234@yahoo.com (H.S.); zhihaocheng1@catas.cn (Z.C.); nweniwinhtet@gmail.com (N.N.W.H.); geyu@catas.cn (Y.G.); 2 Department of Bioinformatics and Biotechnology, Government College University, Faisalabad 38000, Pakistan; usmanijazahmad1246@gmail.com

**Keywords:** MYB transcription factor, expression profile, *Musa acuminata*, *M. balbisiana*, ripening

## Abstract

MYB transcription factors (TFs) make up one of the most important TF families in plants. These proteins play crucial roles in processes related to development, metabolism, and stimulus-response; however, very few studies have been reported for the characterization of MYB TFs from banana. The current study identified 305 and 251 MYB genes from *Musa acuminata* and *Musa balbisiana*, respectively. Comprehensive details of MYBs are reported in terms of gene structure, protein domain, chromosomal localization, phylogeny, and expression patterns. Based on the exon–intron arrangement, these genes were classified into 12 gene models. Phylogenetic analysis of MYBs involving both species of banana, *Oryza sativa*, and *Arabidopsis thaliana* distributed these genes into 27 subfamilies. This highlighted not only the conservation, but also the gain/loss of MYBs in banana. Such genes are important candidates for future functional investigations. The MYB genes in both species exhibited a random distribution on chromosomes with variable densities. Estimation of gene duplication events revealed that segmental duplications represented the major factor behind MYB gene family expansion in banana. Expression profiles of MYB genes were also explored for their potential involvement in acetylene response or development. Collectively, the current comprehensive analysis of MYB genes in both species of banana will facilitate future functional studies.

## 1. Introduction

Transcription factors (TFs) perform an essential role in the regulation of gene expression by suppressing or activating their target genes to control various phases of plant development and growth [1]. MYB TFs are commonly present in fungi, vertebrates, and plants, and make up one of the biggest TF families in plants [2]. The members of the MYB family contain a highly conserved and distinctive N-terminal DNA-binding or protein-protein interaction domain that typically bears one to four imperfect repeats of a particular sequence (termed R1, R2, R3, and R4) and three α-helices. Each repeat comprises almost 52 amino acids in length [3]. The second and third helices form a special structure called a helix–turn–helix (HTH) that binds to the major grooves of DNA [4,5]. However, the C terminal region of the MYB domain is greatly divergent. It acts as an activation domain for the wide-ranging roles of the MYB family [6]. The number of repeats in the MYB domain is used as a basic criterion to classify MYB proteins. In plants, the MYB family is subdivided into four subfamilies, namely, “MYB-related proteins” (1R-MYB), “R2R3-MYB proteins” (2R-MYB), “R1R2R3-MYB proteins” (3R-MYB), and “4R or Atypical MYB protein” (4R-MYB). In higher plants, the MYB members with two repeats (R2R3) are predominantly present [7].

Since the identification of the first MYB gene in plants, research has been broadly conducted in plants to identify and functionally characterize other members of this gene family [2,8]. Numerous MYB proteins are important for the control of several biochemical and physiological processes of plant development, primary/secondary metabolism, and plant response to abiotic and biotic stresses [2,9,10] (Table S1). For example, the betalain pathway is regulated by *BvMYB1* in beets [11]. Several studies have shown that MYB TFs are involved in the activation or repression of genes associated with the production of pigments/anthocyanins in fruits [12]. Furthermore, MYB TFs (FaEOBII and FaMYB10) also initiate the production of volatile compounds during the ripening of strawberry fruit [13]. The conical cell shape development of the epidermal cells is regulated by *DhMYB1* in the flower labellum of *Dendrobium hybrida* [14]. In cotton, *MYB108* is involved in response to *Verticillium dahliae* infection [15]. Moreover, there are several other examples of potential MYB gene involvement in some characteristic traits of plants, including wine-quality formation in grapes, legume-specific nodulations, and pollinator preferences [16,17].

Banana (*Musa acuminata* L.) is an herbaceous plant from monocotyledons that is generally distributed in tropical and subtropical regions. The two species of *Musa* (*M. acuminata* with an AA genome *Musa balbisiana* with a BB genome) belong to the Musaceae family (2n = 22 chromosomes) and represent the two key ancestors of cultivated varieties of banana [18,19]. Despite its role in plant growth, development, cell differentiation, fruit ripening, and stress response, there is an absolute dearth of research focusing on MYB genes in banana plants [18,20,21]. Whole-genome sequencing of *M. acuminata* and *M. balbisiana* has facilitated study of genome-wide evolution and divergence of the gene families in these plants. The present study systematically identified *MYB* genes by evaluating the sequences of two *Musa* genomes (*M. acuminata* and *M. balbisiana*) using a set of bioinformatics tools. The results of this study will give insight into the organization, distribution, and evolution of the MYB gene family. Moreover, the expression pattern of MYB genes will contribute to functional characterization and understanding the biological role of these genes in banana.

## 2. Results and Discussion

### 2.1. In Silico Identification and Sequence Characterization of MYB Family Genes

The MYB domain sequences of *Oryza sativa* and *Arabidopsis thaliana* were employed as a query for the identification of MYB proteins present in the genomes of *M. acuminata* and *M. balbisiana.* The redundant sequences and candidate genes with imperfect open reading frames (ORFs) were discarded and remaining sequences were used for further analysis. For additional confirmation of the MYB domain in identified proteins, the sequences were subjected to Pfam and PROSITE screening. After carefully surveying the *M. acuminata* and *M. balbisiana* MYB proteins and confirming the conserved characteristics of motifs and domains, 305 non-redundant MYBs were predicted in *M. acuminata* and 251 in *M. balbisiana*. These genes were termed as *MaMYB* and *MbMYB* (Table S2). The MYB members have been studied in diverse plant species (Table 1). Given the genome size in both Musa spp., the number of *MYB* proteins is expected to be two to three times larger than *A. thaliana* and similar to *O. sativa*. However, the comparison of genome size and number of MYBs failed to exhibit a clear relationship (Table 1). It has been assumed that multiple genome duplication events introduced universal problems in genomics for the distinction and understanding of ohnologs (missing from orthologs) [22,23]. However, according to the earlier reports, there were three whole-genome duplications (WGDs) in the *Musa* lineage [18,19]. Therefore, it is likely that variation in the number of *MYBs* could be related to WGDs. The insertion of repetitive sequences might lead to the pseudogenization of paralogs. Moreover, the changes in the regulatory and genic sequences could potentially facilitate the inactivation of genes or sequence divergence [22,24]. A classification of MYB genes (based on the presence of one, two, three, and four MYB repeats) indicated that there were 73 MYB-1R or MYB-related, 222 MYB-R2R3, 07 MYB-3R, and 03 MYB-4R MYBs of the respective categories in *M. acuminata.* Similarly, the genome of *M. balbisiana* possessed 59 MYB-1R or MYB-related, 184 MYB-R2R3, five MYB-3R, and three MYB-4R proteins. A comparison of such categories in close relatives of Musa spp. (i.e., *O. sativa* [25] and *B. distachyon* [26] (Figure S1)) showed that there was a comparable number of genes for 1R, 3R, and 4R categories. However, in Muss spp. the number of genes in the R2R3 category was more than two times this amount. A phylogenetic analysis of R2R3-MYBs of 50 eukaryotic organisms [27] suggested that after divergence from a common ancestor the clades could expand distinctively or in a lineage-specific manner.

**Figure 1:**
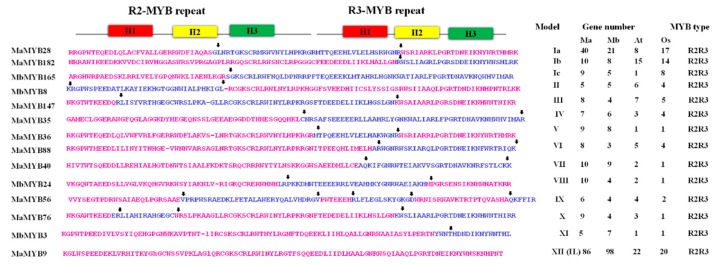
The R2 and R3 repeat coding exon-sequence-based group representation of MYB proteins. The positions of introns are demonstrated with corresponding amino acid residues as arrows, which also represent the position of splicing within the condign sequence. The conserved helices are drawn over the sequences. The numbers of genes in four different plant species are listed on the right side. Intron-less (IL), Ma = *Musa acuminata*, Mb = *Musa balbisiana*, Os = *Oryza sativa,* At = *Arabidopsis thaliana*.

The lengths of predicted proteins varied from 142 to 1125 and 113 to 996 amino acids in *M. acuminata* and *M. balbisiana,* respectively. The relative molecular weight (Mw) ranged between 11.197 and 62.737 kDa in *M. acuminata* and 10.187 and 63.417 in *M. balbisiana*. The predicted theoretical isoelectric point (PI) was 4.71–10.47 in *M. acuminata* and 4.48–11.26 in *M. balbisiana* (Table S2). The MYB proteins of *M. acuminata* and *M. balbisiana* were classified into 12 groups (including an intron-less group) depending on the coding sequence for the structure of R2 and R3 MYB repeats (Figure 1). The MYB proteins from *O. sativa* and *A. thaliana* were also categorized in these 12 models (MI-MXII). According to Model I, the exon-1 of a MYB gene codes for the first two helices of the R2 MYB repeat, the second exon codes for a part of the third helix in R2 domain, and the first helix in R3 repeats. Similarly, exon-3 is responsible for coding the second and third helix of R3. This is represented in Model Ia. Model I even codes for the three helices of R2 and first helix of R3 (Ib), or the first two helices are coded by intron-1 and the remaining helices are coded by intron-2 (Model Ic). Similarly, there are other variations in gene models, as described by Models II to XI. The R2 and R3 repeats of *MaMYB56*-like genes (MYB genes with the most number of introns) were classified in Model IX. All the intron-less genes were grouped into MXII. These findings are in accordance with previous investigations [32] that have explained the evolution of MYB genes in lower and higher plant species from a common pool of multiple MYB genes. The group MXII prospectively constitutes the most ancient MYB types [32].

**Figure 2:**
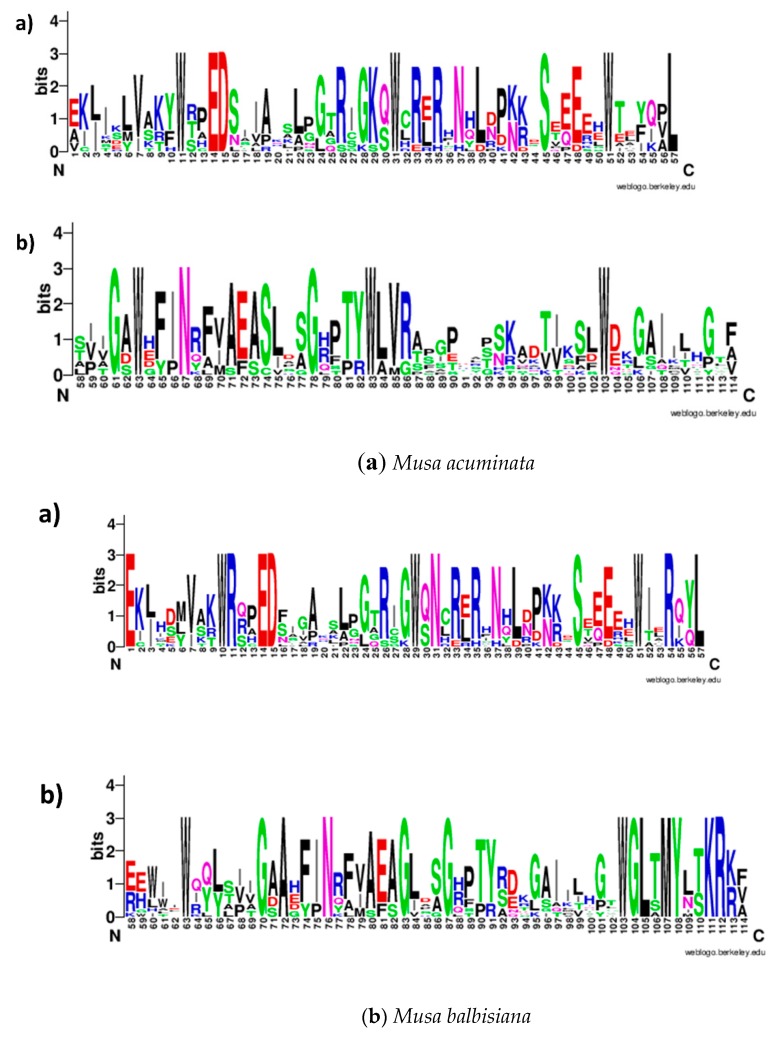
Sequence logos of the conserved R2 and R3 repeats of the MYB domain across all R2R3-MYB proteins in the (**a**) *M. acuminata* and (**b**) *M. balbisiana* genomes. The sequence logos are based on global alignments of R2/R3 MYB repeats from *M. acuminata* and *M. balbisiana* R2R3-MYB domains.

The basic structure of the MYB domain consists of almost 50 amino acids with three regularly spaced Trp (W) residues and three α-helices: H1, H2, and H3 [33]. Consistent with earlier reports, *MaMYB* and *MbMYB* genes harbored the typical MYB domain and contained a characteristic amino acid with a series of highly conserved Trp residues. These residues are considered a landmark of MYB proteins and play a significant role in the sequence-specific binding of DNA (Figure 2). The second Trp residue in the R3MYYB domain of *M. balbisiana* was replaced by other amino acids, which may have affected the binding affinity of the DNA. In addition to highly conserved Trp residues, other highly conserved residues were observed in more than 90% of MYB domains from both *M. acuminata* and *M. balbisiana*.

Introns usually undergo rapid changes and are often neutral to selection during the process of evolution; hence, higher sequence-similarity between orthologous introns show a functional restraint in the evolutionary process [34]. The intron–exon structure of R2R3-MYB repeats in higher plants is conserved, and intron-containing genes have previously been sub-grouped into four to six groups [17,25,35]. In this study, exon–intron organization was examined to better understand the structural organization, which was found to be similar in both banana species. Moreover, it was observed that most of R2R3-MYB genes had 1 to 10 introns in the coding sequence. Similarly, seven of the 1R-MYBs had three introns while six of the R3-MYB genes had four introns (Figure 2a,b). These outcomes exhibit the occurrence of well-preserved configurations within the MaMYB and MbMYB subfamilies and elevated sequence variation amongst diverse groups of the two banana species.

### 2.2. Chromosomal Distribution and Duplication

**Figure 3:**
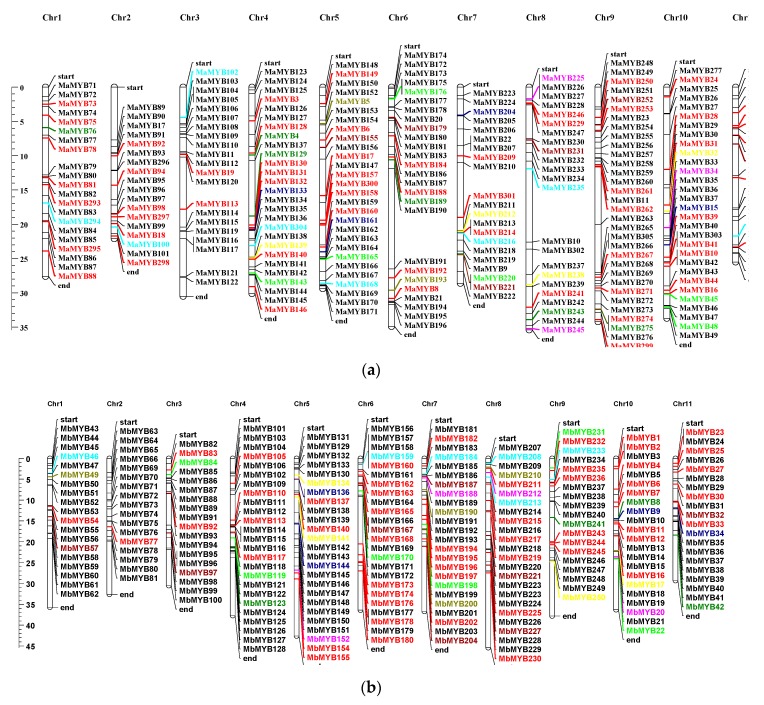
The arrangement of MYB genes on the chromosomes of (**a**) *Musa acuminata* and (**b**) *Musa balbisiana*. The chromosome numeral (Chr) is shown above every chromosome. The arrangement is designated on a centimorgan (cM) scale. The red color represents segmental duplication. Similar colors (other than red) represent the tandem duplicated genes.

The genomic sequences of MYB genes were taken from NCBI, and the chromosomal location of MYB genes on each chromosome was mapped by MapChart. Analysis of genomic location displayed that the banana MYB genes were randomly distributed throughout 11 linkage groups (Figure 3a,b). In *M. acuminata*, out of 305 genes, 283 genes were mapped on 11 chromosomes. Most MaMYB genes were found on chromosome 6 (35 genes), while chromosome 3 had the least number of genes (18). Chromosomal positions of 17 genes (*MaMYB12, MaMYB13, MaMYB14, MaMYB278, MaMYB279, MaMYB280, MaMYB281, MaMYB282, MaMYB283, MaMYB284, MaMYB285, MaMYB286, MaMYB287, MaMYB288, MaMYB289, MaMYB290, MaMYB291*) were unavailable. They were present on the scaffolds, which have not yet assigned to any linkage group (Figure 3a).

Out of 251 genes in *M. balbisiana*, 237 MYB genes were mapped on 11 chromosomes (Figure 3b). Like *M. acuminata*, all MYB genes of *M. balbisiana* were randomly distributed. Most MYB genes were present on chromosome 4 and 5 (25 genes on each). Chromosome 2 carried the least number of MYB genes (17 genes). It is interesting to note that chromosome 2 in both banana species carried the least number of MYB genes (Figure 3a,b). There were 14 MbMYB genes (*MbMYB48, MbMYB67, MbMYB68, MbMYB90, MbMYB107, MbMYB108, MbMYB120, MbMYB135, MbMYB153, MbMYB175, MbMYB205, MbMYB206, MbMYB242, MbMYB251*) present in scaffold regions.

Almost 26% of MaMYB genes were duplicated representing either segmental duplication (51 genes) or tandem duplication (30 genes) (Table S4). Similarly, almost 24% of MbMYB genes experienced duplication segmentally (38) or tandemly (23). The non-synonymous (Ka)/synonymous (Ks) ratio was calculated for all the tandemly and segmentally duplicated *MYB* gene-pairs as less than 1 or greater than 1, respectively. This revealed that the gene pairs with a Ka/Ks ratio of less than 1 were subjected to purifying selection, and those with a ratio of more than 1 experienced positive selection [36,37]. In the evolutionary process, most plants experienced one or more ancient polyploidies. Gene duplication has long been known to occur during plant evolution, thereby contributing to the formation of new gene functions, expansion of large gene families, and origins of evolutionary novelty [38]. The genome *Arabidopsis* has undergone two current whole-genome duplications (WGD; α and β) in the lineage of Brassicaceae [39]. A previous report classified the chromosomal duplication of *Arabidopsis* into three types based on the duplication times of α, β, and γ [39]. Accordingly, whole-genome analysis of the *MYB* gene family in *Glycine max, Populus trichocarpa*, *Oryza sativa,* and *Zea mays* showed that multiple tandem and segmental duplications events play a crucial role in the expansion of the MYB gene family [17]. High segmental and low tandem duplications have commonly been present in the MYB gene family in plants [38], which is supported by several publications [16,38,40]. Copies of genes evolved by segmental duplications are more often reserved in the slowly evolving MYB gene family than in tandem duplication [38]. Higher proportions of segmental duplication has revealed that the expansion of MYB genes in both *M. acuminata* and *M. balbisiana* is due to the segmental duplication [41]. Moreover, a lower tandem gene duplication is potentially associated with gene families involved in housekeeping or key regulatory functions [38,42].

### 2.3. Phylogenetic Analysis of Musa acuminata and Musa balbisiana

**Figure 4:**
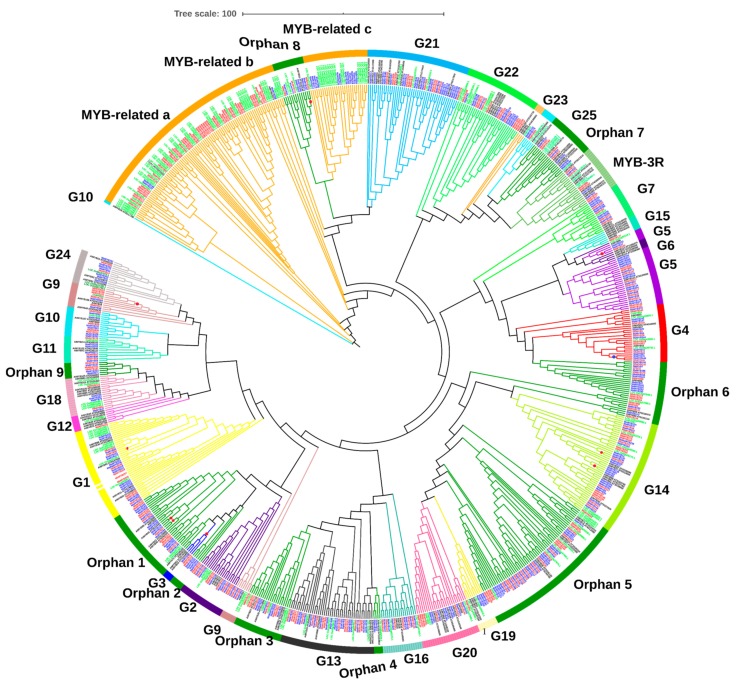
A phylogenetic tree of MYB proteins based on functional studies. MYB genes of *M. acuminata* (Ma, blue), *M. balbisiana* (Mb, red), *O. sativa* (Os, green), and *A. thaliana* (At, black) were used. The amino acid sequence was aligned by CLUSTALW and the phylogenetic tree was made using the neighbor-joining method.

To examine the phylogenetic relationship of banana MYB genes, a phylogenetic tree was constructed from protein sequences of *A. thaliana, O. sativa M. acuminata*, and *M. balbisiana* (Figure 4). The topologies of the phylogenetic tree were similar to previously reported results from a phylogenetic analysis comparing *O. sativa* and *A. thaliana* [25,26,30].

Comparative analysis of MYB genes in these genomes indicated that genes with orthologous relationships happened to be grouped in the phylogenetic tree rather than in paralogs. This reveals that a higher diversification of MYB genes occurred in the ancestor species during the evolutionary process [27,43]. According to our results and based on previously reported analyses of *O. sativa* and *A. thaliana* MYB proteins [25,44], the MYB proteins of *M. acuminata* and *M. balbisiana* could be classified into 25 MYB-R2R3 subgroups (i.e., G1 to G25), nine orphan or atypical subgroups, three MYB-related subgroups (a,b,c), and one MYB-3R subgroup. The phylogenetic analysis showed that there was an unequal representation of members from *M. acuminata*, *M. balbisiana*, *O. sativa*, and *A. thaliana*. For example, G6, G12, and G23 did not contain any member from *M. acuminata* or *M. balbisiana*. This might indicate a loss of corresponding members after divergence [45]. Otherwise, it is possible that they were lost in the assembly or annotation of the banana genome. Moreover, some groups (like atypical 2) included members only from *M. acuminata* and *M. balbisiana* [46]. It has been suggested that such genes might have a specialized role in banana, and that they were either lost in *O. sativa* and *A. thaliana*, or acquired in these fruit species, after divergence from common ancestors [27]. Such groups are good candidates for phylogenetic and functional studies of banana. Similarly, MYBs from both species of banana shared most of the groups except G15, G19, atypical 4, atypical 5, and MYB-related c groups. Members of such groups are important candidates for species-specific functional investigations (Table 2).

The phylogenetic results were consistent with recent reports [26,32,47] showing that various groups included more MYBs from Arabidopsis than *M. acuminata*, *M. balbisiana*, and *O. sativa*. These results are in accordance with the current information that *Arabidopsis* underwent more duplication events after divergence of the last common ancestor from these three close species. This suggests that the existence of species-specific MYBs were either acquired in the Musa lineages after divergence from common ancestors, or lost in *Arabidopsis* [25]. The physiological and anatomical differences between *M. acuminata*, *M. balbisiana*, and *Arabidopsis* strengthen the prediction that some members of the MYB family may have been differentially expanded. Outside and inside of these functional clades, *M. acuminata* and *M. balbisiana* MYB genes appeared as clusters or gene pairs (Figure 4). Moreover, comparative phylogenetic analysis between *M. acuminata* and *M. balbisiana* revealed a high level of conservation between the two genomes of banana.

### 2.4. Expression Profile of MYB Genes during Fruit Ripening

**Figure 5:**
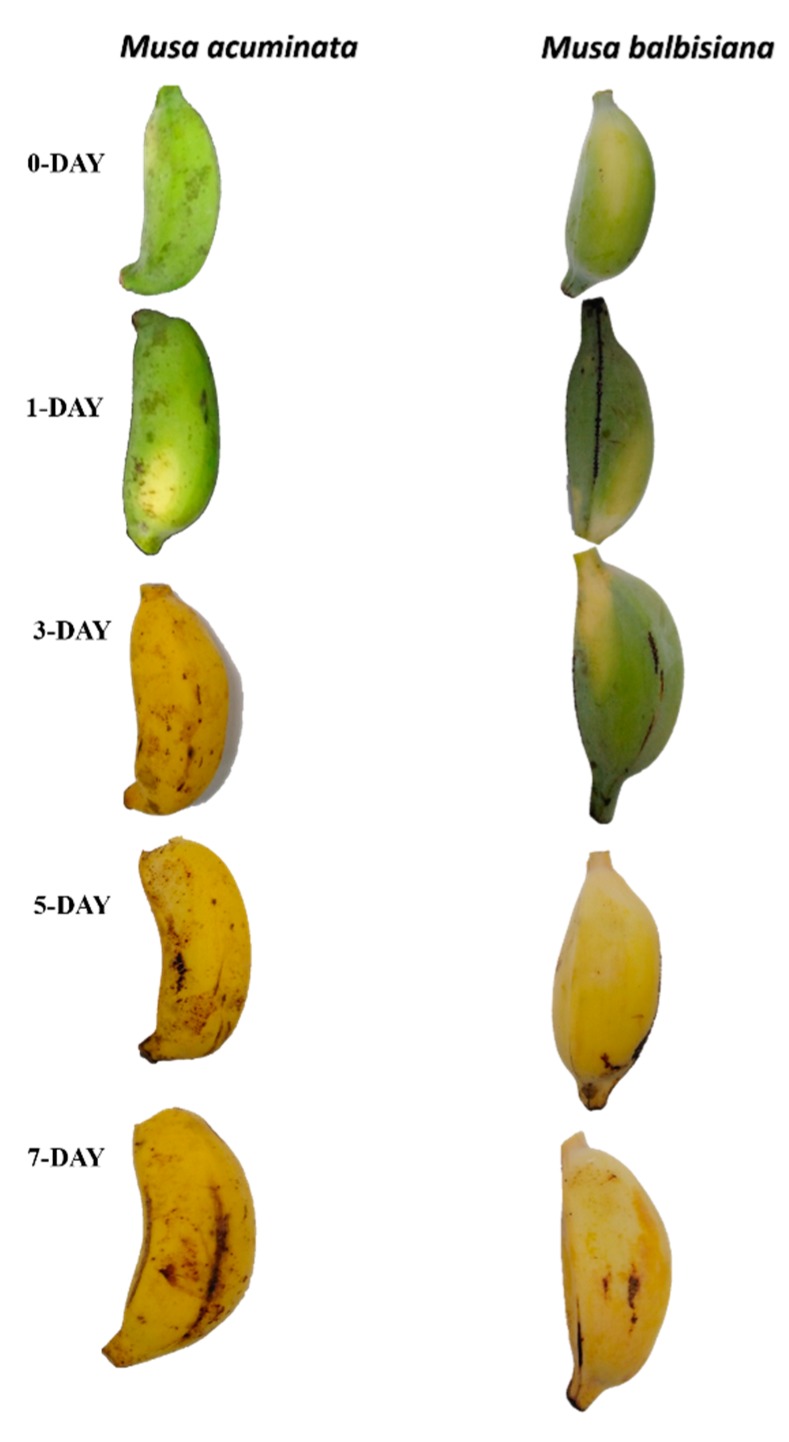
Comparison of *M. acuminata* and *M. balbisiana* fruits at different stages of fruit ripening.

MYB genes have been widely studied and found to be involved in numerous plant-specific processes including development, metabolism, and gene expression regulation for biotic and abiotic stress response [17,33,34,35]. Therefore, to gain more insight into the role of MYB genes in the ripening of banana (Figure 5), relative real-time RT-qPCR was performed to evaluate the transcript abundance of target genes in fruit ripening.

**Figure 6:**
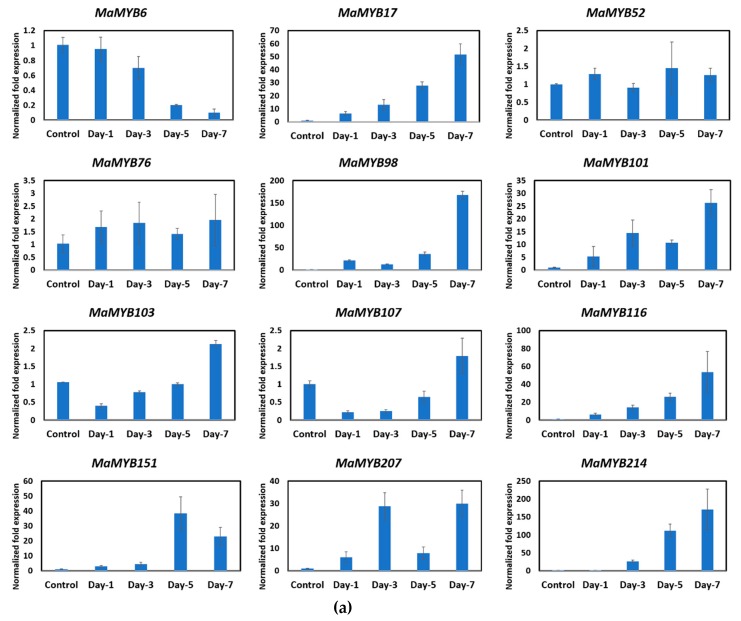

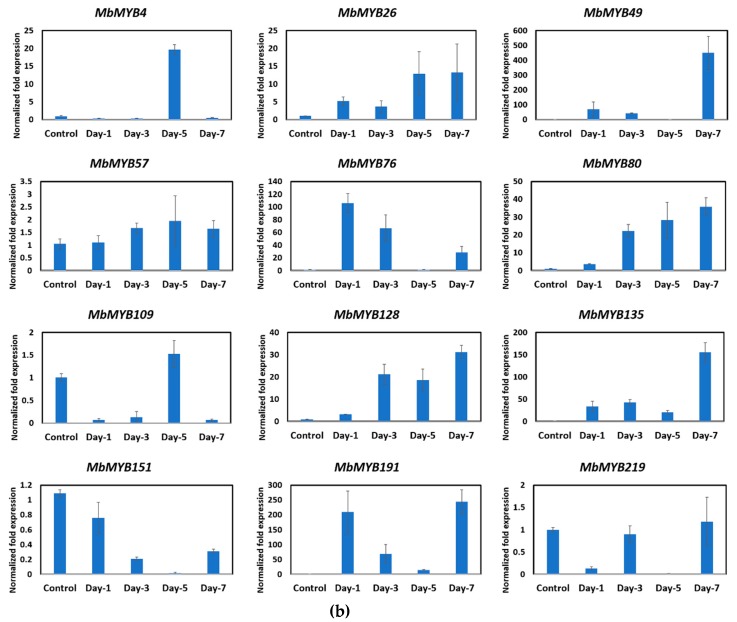
Relative RT-qPCR assay of MYB genes in (**a**) *M. acuminata* and (**b**) *M. balbisiana* during ripening before and after the application of acetylene. The default expression value for each gene was from non-treated plants. Error bars indicate the standard deviation from the mean (three replicates).

After the application of acetylene, both banana species responded differentially. Banana finger-drop was observed in *M. acuminata* after three days. However, it was delayed in *M. balbisiana*, which is considered less sensitive to acetylene application [48]. The RNA-seq data were accessed from the Banana Genome Hub to select MYB genes responsive to acetylene application [49]. For validation, mostly those genes were selected that shared a clade in the phylogenetic tree. These MYB genes (from both species) are strong homologs, as most of them are present on same monophyletic taxon (represented by colored dots in phylogenetic tree). Twelve MYB genes were selected (from each species) for validation through qPCR (Table S3). The results of qPCR were mostly associated with RNA-seq expression data. However, some differences were also found. Although we applied similar growth conditions, such differences could be related to genotypic differences, plant age, or even sampling time in a day. The real-time expression profile showed diverse expression patterns. The transcript abundance of some MYB genes (*MaMYB 17, 98, 101, 116, 151, 207*, and *214*) increased with time (Figure 6a). Similarly, the expression of *MbMYB26, 49, 80, 128, 135*, and *191* was increased, while the expression of *MbMYB76* and *151* was decreased with time (Figure 6b). This suggests a potential involvement of these genes in fruit ripening or development. In the phylogenetic tree, these genes were clustered in G1 (*MaMYB101/MbMYB80*), G3 (*MaMYB98/MbMYB76*), G4 (*MaMYB76/MbMYB49*), G5 (*MaMYB17/MbMYB135*), G9 (*MaMYB214/MbMYB151*), G14 (*MaMYB6/MbMYB128*), as well as in the Atypical/Orphan 1 (*MaMYB151/MbMYB57*) and Atypical/Orphan 8 (MaMYB207/MbMYB191) groups (Figure 4). Members of these groups are involved in several processes related to plant development [44]. In G4, MYB proteins from banana (MaMYB76 and MbMYB49) clustered with AtMYB04 (Figure 4), which is involved in flavonoid biosynthesis [50,51]. Similarly, AtMYB5 (shared clade with MaMYB17 and MbMYB135 in G5) is involved in regulating PA biosynthesis [52,53,54]. The expression of *MaMYB6* decreased from day 0 to 7 (Figure 6a). The *MYB6* is a transcriptional repressor that negatively regulates the ripening of banana fruit by obstructing starch degradation [55]. A progressive downregulation of *MYB6* transcripts correlates with a conserved repressive role of this gene.

## 3. Material and Methods

### 3.1. Sequence Database Searches

The Banana Genome Hub (https://banana-genome-hub.southgreen.fr/) was used to retrieve the sequences of both banana species (i.e., *M. acuminata* and *M. balbisiana*). The protein sequences for MYBs from A. thaliana and O. sativa were obtained from the Arabidopsis Information Resource (TAIR release 10, http://www.arabidopsis.org), Rice Genome Annotation Project database (RGAP release 7, http://rice.plantbiology.msu.edu/index.shtml), and plant transcription factor database (PlantTFDB: http://planttfdb.cbi.pku.edu.cn/). The IDs and names of genes were listed in Table S2.

### 3.2. The Identification and Chromosomal Mapping of MYB TFs

The Pfam database (http://pfam.xfam.org/) was accessed to retrieve the SANT domain (PF00249) representing the MYB binding domain. It was used to screen MYB genes in both species of banana using HMMER (v3.1b2) (http://hmmer.org). An e-value of less than 1e-10 was used as a cut off threshold. For further confirmation of the identity of each sequence as MYB, these sequences were also subjected to a screening in SMART (http://smart.embl-heidelberg.de/) and CDD (http://www.ncbi.nlm.nih.gov/Structure/cdd/wrpsb.cgi). The gene structure display server (GSDS) (http://gsds.cbi.pku.edu.cn/) was used to obtain a schematic representation of all gene structures. The molecular weight ad isoelectric point was investigated through Expasy (http://web.expasy.org/protparam/). The MapChart program (http://www.biometris.wur.nl/UK/Software/MapChart/download) was used to demonstrate the physical location of the MYB genes on chromosomes of *M. acuminata* and *M. balbisiana*. The gene duplication events were determined through the DNAsp program using the rates of non-synonymous (Ka) and synonymous (Ks) substitutions.

### 3.3. Phylogenetic Analysis

For this analysis, multiple sequence alignment was performed through ClustalW in Mega 7.0 software. Later on, a phylogenetic tree was built using the neighbor-joining likelihood method with 1000 bootstrap replicates. MYB protein sequences from *M. balbisiana*, *M. acuminate*, *A. thaliana*, and *O. sativa* were used for the analysis of phylogenetic and evolutionary relationships.

### 3.4. Plant Material and Stress Imposition

Fruits were harvested from *M. acuminata* and *M. balbisiana* at the green maturation stage. The plants were grown in an orchard in Danzhou, Hainan, China. A 500× diluted solution of 30% acetylene (dissolved in ethanol) was applied to the detached fruits. Acetylene is an analog of ethylene, which is used for artificially ripening fruits [56]. It has a lesser biological activity than ethylene and there is no significant difference in sensory attributes between bananas treated with ethylene and acetylene [57]. Acetylene was applied for one, three, five, and seven days after washing the fruits with clean water. The control samples were collected before treatment. The treated and non-treated fruits were kept at 22 °C under dark conditions.

### 3.5. RNA Extraction and Quantitative Real-Time PCR (qRT-PCR)

An RNA extraction Kit (RNAprep Pur Plant Kit for polysaccharides and polyphenolic-rich samples from TIANGEN Biotech, Beijing) was used for total RNA extraction from banana fruits according to the instruction of the manufacturer. RNA was quantified using Thermo Nanodrop 2000 (Thermo Fisher Scientific, Massachusetts). One microgram of RNA was reverse-transcribed for cDNA synthesis using an All-in-One First-Strand Synthesis kit (Monad, Jiangsu, China cDNA). The cDNA was stored at −20 °C for further use. The qRT-PCR analysis was performed using an Applied Biosystems StepOnePlus Real-Time PCR System and TB Green^T^ Premix Ex Taq^T^ II, Tli RNaseH plus kit (Takara). The Oligo Calculator tool (http://mcb.berkeley.edu/labs/krantz/tools/oligocalc.html) was used to design gene-specific primers, and specificity of primers was verified by NCBI-primer BLAST program (https://www.ncbi.nlm.nih.gov/tools/primer-blast/) (Table S3). Expression analysis of MYB genes was repeated three times and the ribosomal protein S (RSP2) was used as the house-keeping gene [58].

## 4. Conclusions

Banana is globally consumed and exhibits a vital role in food security for millions of people around the world. The MYB genes of *M. acuminata* and *M. balbisiana* were classified into 12 groups (MI-MXII) based on intron and exon structure models of the MYB domain sequences. MaMYB and MbMYB genes were randomly distributed on 11 chromosomes. Phylogenetic analysis of the MYB family among banana and plant species models indicated the functional divergence during evolution. The current study reports the existence of species-specific MYBs that are potential candidates for functional characterization. Of the two species, *M. acuminata* seemed more sensitive to acetylene than *M. balbisiana* did. The transcript abundance of *MaMYB*s (i.e., *MaMYB17, 98, 101, 116, 151, 207*, and *214*) and *MbMYBs (MbMYB26, 49, 80, 128, 135*, and *191*) was increased in response to acetylene or development. The findings of this study will facilitate future studies aimed at the functional characterization of MYB TFs in banana.

## Figures and Tables

**Table 1:** MYB transcription factors identified in plant species of diverse genome sizes.

Sr#	Name of Plant	Genome Size (Mbs) *	Number of MYBs	Reference
1	*Arabidopsis thaliana*	119	197	[25]
2	*Sesamum indicum*	270	278	[28]
3	*Brachypodium distachyon*	270	122	[26]
4	*Citrus sinensis*	301	177	[22]
5	*Oryza sativa*	373	155	[25]
6	*Musa acuminata*	332	305	Current study
7	*Musa balbisiana*	430	251	Current study
8	*Physcomitrella patens*	467	116	[29]
9	*Solanum tuberosum*	663	158	[30]
10	*Solanum lycopersicum*	827	127	[31]

* Taken from the National Center for Biotechnology Information (NCBI) genome database.

**Table 2:** Details of the number of genes in each group of phylogenetic trees for each species understudy.

Short Name	MaMYB	MbMYB	AtMYB	OsMYB
G1	16	10	5	9
G2	7	8	3	3
G3	1	1	2	0
G4	10	8	4	4
G5	17	11	2	0
G6	0	0	4	0
G7	8	4	3	1
G9	3	4	3	1
G10	5	3	2	1
G11	5	2	3	1
G12	0	0	7	0
G13	27	14	9	11
G14	23	15	6	7
G15	0	1	5	0
G16	7	6	3	2
G18	4	2	5	5
G19	4	0	3	2
G20	9	8	6	3
G21	23	11	9	3
G22	12	11	4	6
G23	0	0	3	1
G24	7	2	3	3
G25	3	4	5	3
Atypical	11	7	3	6
Atypical 2	4	2	0	0
Atypical 3	7	6	4	5
Atypical 4	2	0	1	1
Atypical 5	0	2	1	2
Atypical 6	6	22	6	9
Atypical 7	13	11	3	2
Atypical 8	5	4	7	4
MYB related a	2	4	1	3
MYB related b	4	3	1	24
MYB related c	8	0	0	17
MYB-3R	3	6	0	17

## Supplementary Materials

The following are available online at https://www.mdpi.com/2223-7747/9/4/413/s1, Figure S1: Phylogenetic tree of plant species., Table S1: Details MYB genes studied in different plant species. Table S2: Gene Ids and their sequence features of MYB genes. Table S3: List of primers used in this study. Table S4: Segmental and tandem duplication in MYB genes in *Musa acuminata* and *Musa balbisiana*.
