# Supplementary material for: Genome-Wide Identification and Comparative Analysis of MYB Transcription Factor Family in Musa acuminata and Musa balbisiana
Source: Plants (Basel). 2020 Mar 27;9(4):413. doi: 10.3390/plants9040413 (PMC7238746; doi:10.3390/plants9040413)
Supplement: Supplementary file 1 [file plants-09-00413-s001.zip › spm-plants-713656-revise1/Table S1-Details MYB genes studied in different plant species.docx]

**Table S1:** Details of MYB genes studied in different plant species

| Serial # | Name of *MYB* gene | Specie | Function | Reference |
| --- | --- | --- | --- | --- |
|  | **Role in Plant development** |  |  |  |
| 1 | At*MYB*021/ At*MYB*033/ At*MYB*065/ | Arabidopsis thaliana | Stamen development/Anther development (tapetum), Filament length, GA– and JA–mediated | (Mandaokar *et al*. [2006](https://link.springer.com/article/10.1007/s12298-013-0179-1#CR76)) |
| 2 | At*MYB*037/ At*MYB*038 | Arabidopsis thaliana | Axillary meristem regulation/Lateral organ formation (shoot branching, GA–mediated) | (Lee *et al*. [2009](https://link.springer.com/article/10.1007/s12298-013-0179-1#CR61)) |
| 3 | At*MYB*068/ At*MYB*084 | Arabidopsis thaliana | Root elongation, Axillary meristem regulation/ Lateral organ formation | (Fang *et al*.,2017) |
| 4 | Os*MYB*2P-1 | Oryza sativa | Root system architecture | (Dai *et al*. [2012](https://link.springer.com/article/10.1007/s12298-013-0179-1#CR19) |
| 5 | Gm*MYB*-G20-1 | Glycine max | Flower color | (Takahashi *et al*. [2013](https://link.springer.com/article/10.1007/s12298-013-0179-1#CR131)) |
| 6 | At*MYB*115/ At*MYB*118 | Arabidopsis thaliana | Embryogenesis | (Wang *et al*. 2009) |
| 7 | Am*MYB*Mx | Antirrhinum majus | Cell shape | (Agarwal,2016) |
| 9 | Ph*MYB*1 | Petunia hybrida | Petal development | (Colanero *et al*.,2018) |
| 10 | At*MYB*016 | Arabidopsis thaliana | Petal development | (Baumann *et al*. [2007](https://link.springer.com/article/10.1007/s12298-013-0179-1#CR4)) |
|  | **Cellular proliferation and differentiation** |  |  |  |
| 11 | At*MYB*005 | Arabidopsis thaliana | Seed coat differentiation | (Gonzalez *et al*. [2009](https://link.springer.com/article/10.1007/s12298-013-0179-1#CR36)) |
| 12 | At*MYB*017 | Arabidopsis thaliana | Early inflorescence development and seed germination | (Zhang *et al*. [2009](https://link.springer.com/article/10.1007/s12298-013-0179-1#CR150)) |
| 13 | At*MYB*046/ At*MYB*057 | Arabidopsis thaliana | Expression in siliques/ flower buds | (Matías‐Hernández *et al*.,2017) |
| 14 | Gh*MYB*109 | Gossypium hirsutum | Fibre elongation | (Suo *et al*. [2003](https://link.springer.com/article/10.1007/s12298-013-0179-1#CR129)) |
|  | **Trichome development** |  |  |  |
| 15 | At*MYB*011 | Arabidopsis thaliana | Trichome formation | (Tian *et al*.,2017) |
| 16 | At*MYB*017/ At*MYB*023 | Arabidopsis thaliana | Cell fate/Trichome initiation and branching, Root hair patterning | Kang *et al*. [2009](https://link.springer.com/article/10.1007/s12298-013-0179-1#CR55) |
| 17 | At*MYB*066 | Arabidopsis thaliana | Root hair development | (Zhou *et al*.,2018) |
|  | **Phenylpropanoid metabolism** |  |  |  |
| 18 | Am*MYB*305/ Am*MYB*340 | Antirrhinum majus | Phenylpropanoid metabolism | (Naing *et al*.,2018) |
| 19 | At*MYB*003 | Arabidopsis thaliana | Phenylpropanoid metabolism | (Dubos *et al*. [2008](https://link.springer.com/article/10.1007/s12298-013-0179-1#CR26)) |
| 20 | At*MYB*011/At*MYB*012 | Arabidopsis thaliana | Phenylpropanoid pathway/ Flavonol biosynthesis | (Stracke *et al*. [2007](https://link.springer.com/article/10.1007/s12298-013-0179-1#CR126)) |
| 21 | At*MYB*075/At*MYB*090 | Arabidopsis thaliana | Phenylpropanoid pathway | (Gonzalez *et al*. [2008](https://link.springer.com/article/10.1007/s12298-013-0179-1#CR35)) |
| 22 | At*MYB*113/ At*MYB*114 | Arabidopsis thaliana | Anthocyanin biosynthesis | (Gonzalez *et al*. [2008](https://link.springer.com/article/10.1007/s12298-013-0179-1#CR35)) |
| 23 | Es*MYB* | E. sagittatum | Flavonoid biosynthesis | (Huang *et al*. [2013](https://link.springer.com/article/10.1007/s12298-013-0179-1#CR44)) |
| 24 | Oj*MYB*1 | *Oenanthe javanica* | Anthocyanin biosynthesis | (Feng *et al*.,2018) |
| 25 | Ph*MYB*3 | Petunia hybrida | Anthocyanin synthesis | (Peng *et al*.,2016) |
| 26 | Ph*MYB*An2 | Petunia hybrida | Anthocyanin synthesis | (Pérez-Díaz *et al*.,2016) |
| 27 | Zm*MYB*C1 | Zea mays | Anthocyanin synthesis | (Li *et al*.,2015) |
| 28 | Zm*MYB*P1 | Zea mays | Anthocyanin synthesis | Grotewold *et al*. [1994](https://link.springer.com/article/10.1007/s12298-013-0179-1#CR37); Du *et al*. [2012](https://link.springer.com/article/10.1007/s12298-013-0179-1#CR25) |
|  | **Hormone Responses** |  |  |  |
| 29 | At*MYB*33/ At*MYB*65 | Arabidopsis thaliana | Hormone response (GA signaling) | (Roy *et al*.,2016) |
| 30 | At*MYB*101 | Arabidopsis thaliana | Hormone response (GA signaling) | (Fang *et al*.,2018) |
| 31 | Hv*MYB*Ga | Hordeum vulgare | Hormone response (GA signaling) | (Butt *et al*.,2017) |
| 32 | OsGA*MYB* | Oryza sativa | Hormone response (GA signaling) | (Zhu *et al*.,2015) |
|  | **Abiotic Stress** |  |  |  |
| 33 | At*MYB*002 | Arabidopsis thaliana | Drought response | Fang *et al*.,2018 |
| 34 | At*MYB*015 | Arabidopsis thaliana | Cold stress tolerance | (Agarwal *et al*. [2006](https://link.springer.com/article/10.1007/s12298-013-0179-1#CR2)) |
| 35 | Os*MYB*4 | Oryza sativa | Cold Stress | (Zhu *et al*.,2015) |
| 36 | Mdo*MYB*121 | Malus | Drought and cold stress | (Erpen *et al*.,2018) |
| 37 | At*MYB*030 | Arabidopsis thaliana | Abiotic stress response, SA–mediated pathway | (Li *et al*. [2009](https://link.springer.com/article/10.1007/s12298-013-0179-1#CR64)) |
| 38 | At*MYB*060/ At*MYB*094 | Arabidopsis thaliana | Drought, ABA–mediated (stomatal closure) | (Cominelli *et al*. [2005](https://link.springer.com/article/10.1007/s12298-013-0179-1#CR15)) |
| 39 | At*MYB*070/ At*MYB*073/ At*MYB*077 | Arabidopsis thaliana | biotic stress response/ Drought, Light, Wounding | (Jung *et al*. [2008](https://link.springer.com/article/10.1007/s12298-013-0179-1#CR54)) |
| 40 | At*MYB*096 | Arabidopsis thaliana | Drought tolerance (ABA and JA–mediated) | (Seo *et al*. [2009](https://link.springer.com/article/10.1007/s12298-013-0179-1#CR115)) |
| 41 | Bc*MYB*1 | Boea crassifolia | Drought tolerance | (Chen *et al*. [2005](https://link.springer.com/article/10.1007/s12298-013-0179-1#CR10)) |
| 42 | Os*MYB*55 | Oryza sativa | Heat stress tolerance | El-kereamy *et al*. [2012](https://link.springer.com/article/10.1007/s12298-013-0179-1#CR28) |
| 43 | Sc*MYB*AS1 | Saccharum officinarum | Drought and salt tolerance | (Prabu and Theertha [2011](https://link.springer.com/article/10.1007/s12298-013-0179-1#CR97), Prabu and Prasad [2012](https://link.springer.com/article/10.1007/s12298-013-0179-1#CR96)) |
|  | **Biotic Stress** |  |  |  |
| 44 | At*MYB*030 | Arabidopsis thaliana | Hypocotyl elongation, brassinosteroid pathway | (Li *et al*. [2009](https://link.springer.com/article/10.1007/s12298-013-0179-1#CR64); Segarra *et al*. [2009](https://link.springer.com/article/10.1007/s12298-013-0179-1#CR112)) |
| 45 | At*MYB*44 | Arabidopsis thaliana | Plant defense response against aphid | (Liu *et al*. [2010](https://link.springer.com/article/10.1007/s12298-013-0179-1#CR71)) |
| 46 | At*MYB*060/ At*MYB*094/ At*MYB*096 | Arabidopsis thaliana | Biotic stress response | (Cominelli *et al*. [2005](https://link.springer.com/article/10.1007/s12298-013-0179-1#CR15); Seo and Park [2010](https://link.springer.com/article/10.1007/s12298-013-0179-1#CR114)) |
|  | **Light response** |  |  |  |
| 47 | Am*MYB*305 | Antirrhinum majus | UV light response | (Liu *et al*.,2017) |
| 48 | At*MYB*004 | Arabidopsis thaliana | UV light response | (Jiang *et al*.,2016) |
| 49 | Pc*MYB*1 | Petroselinum crispum | Light response | (Shi & Xie *et al*.,2104) |
|  | **Nutrient deficiency** |  |  |  |
| 50 | At*MYB*28 | Arabidopsis thaliana | Sulfur-starvation response | (Naing & Kim ,2018) |
| 51 | At*MYB*29 | Arabidopsis thaliana | Sulfur-starvation response | (Hirai *et al*. [2007](https://link.springer.com/article/10.1007/s12298-013-0179-1#CR42)) |
|  | **Regulation of primary and secondary metabolism** |  |  |  |
| 52 | At*MYB*058/ At*MYB*063 | Arabidopsis thaliana | Lignin biosynthesis (fibers and vessels) | (Zhou *et al*. [2009](https://link.springer.com/article/10.1007/s12298-013-0179-1#CR154)) |
| 53 | At*MYB*123 | Arabidopsis thaliana | Proanthocyanidins (PAs) biosynthesis | (Lepiniec *et al*. [2006](https://link.springer.com/article/10.1007/s12298-013-0179-1#CR62), (Colanero *et al*.,2018) |
| 54 | At*MYB*028/At*MYB*034/ At*MYB*122 | Arabidopsis thaliana | Glucosinolate biosynthesis | (Gigolashvili *et al*. [2007](https://link.springer.com/article/10.1007/s12298-013-0179-1#CR32),) |
| 55 | At*MYB*052/At*MYB*054/ At*MYB*069 | Arabidopsis thaliana | Cell wall thickening (fibers) | (Zhong *et al*. [2008](https://link.springer.com/article/10.1007/s12298-013-0179-1#CR153)) |

**References:**

Agarwal, T. R. (2016). *Syntelogs of MYB31 and MYB42 exhibit divergent roles in phenylpropanoid pathway regulation in maize, sorghum, and rice* (Doctoral dissertation, University of Toledo).

Baumann, K., Perez-Rodriguez, M., Bradley, D., Venail, J., Bailey, P., Jin, H., & Martin, C. (2007). Control of cell and petal morphogenesis by R2R3 MYB transcription factors. *Development*, *134*(9), 1691-1701.

Butt, H. I., Yang, Z., Gong, Q., Chen, E., Wang, X., Zhao, G., & Li, F. (2017). GaMYB85, an R2R3 MYB gene, in transgenic Arabidopsis plays an important role in drought tolerance. *BMC plant biology*, *17*(1), 142.

Chen, B. J., Wang, Y., Hu, Y. L., Wu, Q., & Lin, Z. P. (2005). Cloning and characterization of a drought-inducible MYB gene from Boea crassifolia. *Plant Science*, *168*(2), 493-500.

Colanero, S., Perata, P., & Gonzali, S. (2018). The atroviolacea gene encodes an R3-MYB protein repressing anthocyanin synthesis in tomato plants. *Frontiers in plant science*, *9*.

Colanero, S., Perata, P., & Gonzali, S. (2018). The atroviolacea gene encodes an R3-MYB protein repressing anthocyanin synthesis in tomato plants. *Frontiers in plant science*, *9*.

Cominelli, E., Galbiati, M., Vavasseur, A., Conti, L., Sala, T., Vuylsteke, M., & Tonelli, C. (2005). A guard-cell-specific MYB transcription factor regulates stomatal movements and plant drought tolerance. *Current biology*, *15*(13), 1196-1200.

Dai, X., Wang, Y., Yang, A., & Zhang, W. H. (2012). OsMYB2P-1, a R2R3 MYB transcription factor, is involved in regulation of phosphate-starvation responses and root architecture in rice. *Plant Physiology*, pp-112.

Dubos, C., Le Gourrierec, J., Baudry, A., Huep, G., Lanet, E., Debeaujon, I., ... & Lepiniec, L. (2008). MYBL2 is a new regulator of flavonoid biosynthesis in Arabidopsis thaliana. *The Plant Journal*, *55*(6), 940-953.

El-Kereamy, A., Bi, Y. M., Ranathunge, K., Beatty, P. H., Good, A. G., & Rothstein, S. J. (2012). The rice R2R3-MYB transcription factor OsMYB55 is involved in the tolerance to high temperature and modulates amino acid metabolism. *PLoS One*, *7*(12), e52030.

Erpen, L., Devi, H. S., Grosser, J. W., & Dutt, M. (2018). Potential use of the DREB/ERF, MYB, NAC and WRKY transcription factors to improve abiotic and biotic stress in transgenic plants. *Plant Cell, Tissue and Organ Culture (PCTOC)*, *132*(1), 1-25.

Fang, Q., Jiang, T., Xu, L., Liu, H., Mao, H., Wang, X., & Yang, L. (2017). A salt-stress-regulator from the Poplar R2R3 MYB family integrates the regulation of lateral root emergence and ABA signaling to mediate salt stress tolerance in Arabidopsis. *Plant Physiology and Biochemistry*, *114*, 100-110.

Fang, Q., Wang, Q., Mao, H., Xu, J., Wang, Y., Hu, H., & Wang, X. (2018). AtDIV2, an RR-type MYB transcription factor of Arabidopsis, negatively regulates salt stress by modulating ABA signaling. *Plant cell reports*, *37*(11), 1499-1511.

Feng, C., Andreasson, E., Maslak, A., Mock, H. P., Mattsson, O., & Mundy, J. (2004). Arabidopsis MYB68 in development and responses to environmental cues. *Plant Science*, *167*(5), 1099-1107.

Feng, K., Xu, Z. S., Que, F., Liu, J. X., Wang, F., & Xiong, A. S. (2018). An R2R3-MYB transcription factor, OjMYB1, functions in anthocyanin biosynthesis in Oenanthe javanica. *Planta*, *247*(2), 301-315.

Gigolashvili, T., Engqvist, M., Yatusevich, R., Müller, C., & Flügge, U. I. (2008). HAG2/MYB76 and HAG3/MYB29 exert a specific and coordinated control on the regulation of aliphatic glucosinolate biosynthesis in Arabidopsis thaliana. *New Phytologist*, *177*(3), 627-642.

Gonzalez, A., Mendenhall, J., Huo, Y., & Lloyd, A. (2009). TTG1 complex MYBs, MYB5 and TT2, control outer seed coat differentiation. *Developmental biology*, *325*(2), 412-421.

Gonzalez, A., Zhao, M., Leavitt, J. M., & Lloyd, A. M. (2008). Regulation of the anthocyanin biosynthetic pathway by the TTG1/bHLH/Myb transcriptional complex in Arabidopsis seedlings. *The Plant Journal*, *53*(5), 814-827.

Grotewold, E., Drummond, B. J., Bowen, B., & Peterson, T. (1994). The myb-homologous P gene controls phlobaphene pigmentation in maize floral organs by directly activating a flavonoid biosynthetic gene subset. *Cell*, *76*(3), 543-553.

Hirai, M. Y., Sugiyama, K., Sawada, Y., Tohge, T., Obayashi, T., Suzuki, A., & Goda, H. (2007). Omics-based identification of Arabidopsis Myb transcription factors regulating aliphatic glucosinolate biosynthesis. *Proceedings of the National Academy of Sciences*, *104*(15), 6478-6483.

Huang, W., Sun, W., Lv, H., Xiao, G., Zeng, S., & Wang, Y. (2013). Isolation and molecular characterization of thirteen R2R3-MYB transcription factors from Epimedium sagittatum. *International journal of molecular sciences*, *14*(1), 594-610.

Jiang, M., Ren, L., Lian, H., Liu, Y., & Chen, H. (2016). Novel insight into the mechanism underlying light-controlled anthocyanin accumulation in eggplant (Solanum melongena L.). *Plant Science*, *249*, 46-58.

Jung, C., Seo, J. S., Han, S. W., Koo, Y. J., Kim, C. H., Song, S. I., & Cheong, J. J. (2008). Overexpression of AtMYB44 enhances stomatal closure to confer abiotic stress tolerance in transgenic Arabidopsis. *Plant physiology*, *146*(2), 623-635.

Kang, Y. H., Kirik, V., Hulskamp, M., Nam, K. H., Hagely, K., Lee, M. M., & Schiefelbein, J. (2009). The MYB23 gene provides a positive feedback loop for cell fate specification in the Arabidopsis root epidermis. *The Plant Cell*, *21*(4), 1080-1094.

Lee, D. K., Geisler, M., & Springer, P. S. (2009). LATERAL ORGAN FUSION1 and LATERAL ORGAN FUSION2 function in lateral organ separation and axillary meristem formation in Arabidopsis. *Development*, *136*(14), 2423-2432.

Lepiniec, L., Debeaujon, I., Routaboul, J. M., Baudry, A., Pourcel, L., Nesi, N., & Caboche, M. (2006). Genetics and biochemistry of seed flavonoids. *Annu. Rev. Plant Biol.*, *57*, 405-430.

Li, C., Ng, C. K. Y., & Fan, L. M. (2015). MYB transcription factors, active players in abiotic stress signaling. *Environmental and Experimental Botany*, *114*, 80-91.

Li, L., Yu, X., Thompson, A., Guo, M., Yoshida, S., Asami, T., & Yin, Y. (2009). Arabidopsis MYB30 is a direct target of BES1 and cooperates with BES1 to regulate brassinosteroid‐induced gene expression. *The Plant Journal*, *58*(2), 275-286.

Liu, R., Lü, B., Wang, X., Zhang, C., Zhang, S., Qian, J., & Dong, H. (2010). Thirty-seven transcription factor genes differentially respond to a harpin protein and affect resistance to the green peach aphid in Arabidopsis. *Journal of Biosciences*, *35*(3), 435-450.

Liu, Y., Wang, L., Zhang, J., Yu, B., Wang, J., & Wang, D. (2017). The MYB transcription factor StMYBA1 from potato requires light to activate anthocyanin biosynthesis in transgenic tobacco. *Journal of Plant Biology*, *60*(1), 93-101.

Mandaokar, A. (2009). MYB108 acts together with MYB24 to regulate jasmonate-mediated stamen maturation in Arabidopsis. *Plant Physiology*, *149*(2), 851-862.

Matías‐Hernández, L., Jiang, W., Yang, K., Tang, K., Brodelius, P. E., & Pelaz, S. (2017). Aa MYB 1 and its orthologue At MYB 61 affect terpene metabolism and trichome development in Artemisia annua and Arabidopsis thaliana. *The Plant Journal*, *90*(3), 520-534.

Naing, A. H., & Kim, C. K. (2018). Roles of R2R3-MYB transcription factors in transcriptional regulation of anthocyanin biosynthesis in horticultural plants. *Plant molecular biology*, 1-18.

Naing, A. H., & Kim, C. K. (2018). Roles of R2R3-MYB transcription factors in transcriptional regulation of anthocyanin biosynthesis in horticultural plants. *Plant molecular biology*, 1-18.

Peng, X., Liu, H., Wang, D., & Shen, S. (2016). Genome-wide identification of the Jatropha curcas MYB family and functional analysis of the abiotic stress responsive gene JcMYB2. *BMC genomics*, *17*(1), 251.

Pérez-Díaz, J. R., Pérez-Díaz, J., Madrid-Espinoza, J., González-Villanueva, E., Moreno, Y., & Ruiz-Lara, S. (2016). New member of the R2R3-MYB transcription factors family in grapevine suppresses the anthocyanin accumulation in the flowers of transgenic tobacco. *Plant molecular biology*, *90*(1-2), 63-76.

Prabu, G. R. (2011). Structure of DNA Binding MYB Transcription Factor Protein (ScMYBAS1-3) from Sugarcane Threading and Ab Initio Modelling. *Journal of Phytology*.

Prabu, G., & Prasad, D. T. (2012). Functional characterization of sugarcane MYB transcription factor gene promoter (PScMYBAS1) in response to abiotic stresses and hormones. *Plant cell reports*, *31*(4), 661-669

Roy, S. (2016). Function of MYB domain transcription factors in abiotic stress and epigenetic control of stress response in plant genome. *Plant signaling & behavior*, *11*(1), e1117723.

Segarra, G., Van der Ent, S., Trillas, I., & Pieterse, C. M. J. (2009). MYB72, a node of convergence in induced systemic resistance triggered by a fungal and a bacterial beneficial microbe. *Plant Biology*, *11*(1), 90-96.

Seo, P. J., & Park, C. M. (2009). Auxin homeostasis during lateral root development under drought condition. *Plant signaling & behavior*, *4*(10), 1002-1004.

Shi, M. Z., & Xie, D. Y. (2014). Biosynthesis and metabolic engineering of anthocyanins in Arabidopsis thaliana. *Recent patents on biotechnology*, *8*(1), 47-60.

Takahashi, R., Yamagishi, N., & Yoshikawa, N. (2012). A MYB transcription factor controls flower color in soybean. *Journal of Heredity*, *104*(1), 149-153.

Tian, N., Liu, F., Wang, P., Zhang, X., Li, X., & Wu, G. (2017). The molecular basis of glandular trichome development and secondary metabolism in plants. *Plant Gene*, *12*, 1-12.

Wang, X., Niu, Q. W., Teng, C., Li, C., Mu, J., Chua, N. H., & Zuo, J. (2009). Overexpression of PGA37/MYB118 and MYB115 promotes vegetative-to-embryonic transition in Arabidopsis. *Cell research*, *19*(2), 224.

Zhang, Y., Cao, G., Qu, L. J., & Gu, H. (2009). Characterization of Arabidopsis MYB transcription factor gene AtMYB17 and its possible regulation by LEAFY and AGL15. *Journal of Genetics and Genomics*, *36*(2), 99-107.

Zhong, R., Lee, C., Zhou, J., McCarthy, R. L., & Ye, Z. H. (2008). A battery of transcription factors involved in the regulation of secondary cell wall biosynthesis in Arabidopsis. *The Plant Cell*, *20*(10), 2763-2782.

Zhou, H., Lin‐Wang, K., Wang, F., Espley, R. V., Ren, F., Zhao, J., & Han, Y. (2018). Activator‐type R2R3‐MYB genes induce a repressor‐type R2R3‐MYB gene to balance anthocyanin and proanthocyanidin accumulation. *New Phytologist*.

Zhou, J., Lee, C., Zhong, R., & Ye, Z. H. (2009). MYB58 and MYB63 are transcriptional activators of the lignin biosynthetic pathway during secondary cell wall formation in Arabidopsis. *The Plant Cell*, *21*(1), 248-266.

Zhu, N., Cheng, S., Liu, X., Du, H., Dai, M., Zhou, D. X., & Zhao, Y. (2015). The R2R3-type MYB gene OsMYB91 has a function in coordinating plant growth and salt stress tolerance in rice. *Plant Science*, *236*, 146-156.
