# Supplementary figures and images for: Genome-Wide Identification and Comparative Analysis of MYB Transcription Factor Family in Musa acuminata and Musa balbisiana
Source: Plants (Basel). 2020 Mar 27;9(4):413. doi: 10.3390/plants9040413 (PMC7238746; doi:10.3390/plants9040413)

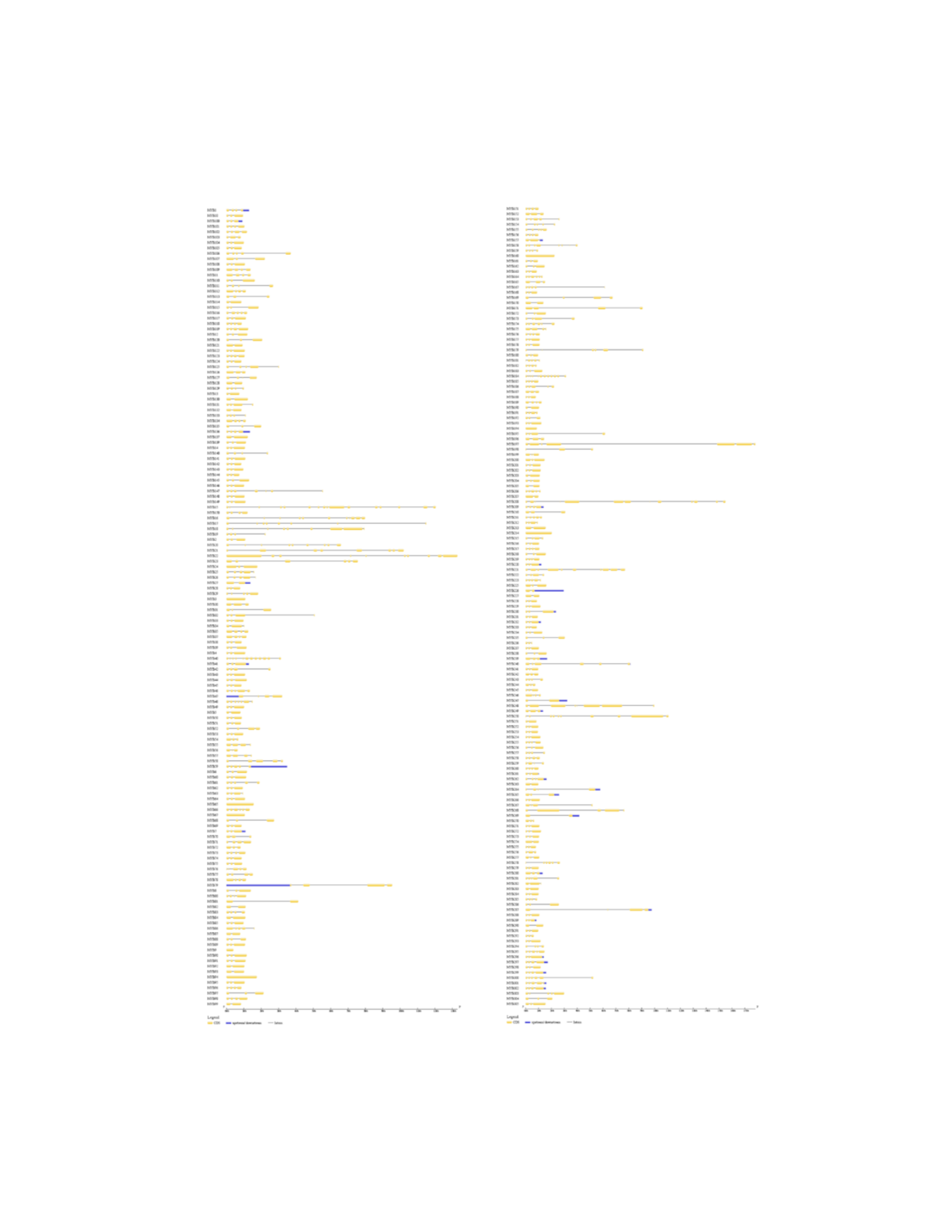

Supplement: Supplementary file 1 [file plants-09-00413-s001.zip › spm-plants-713656-revise1/S2.tif]

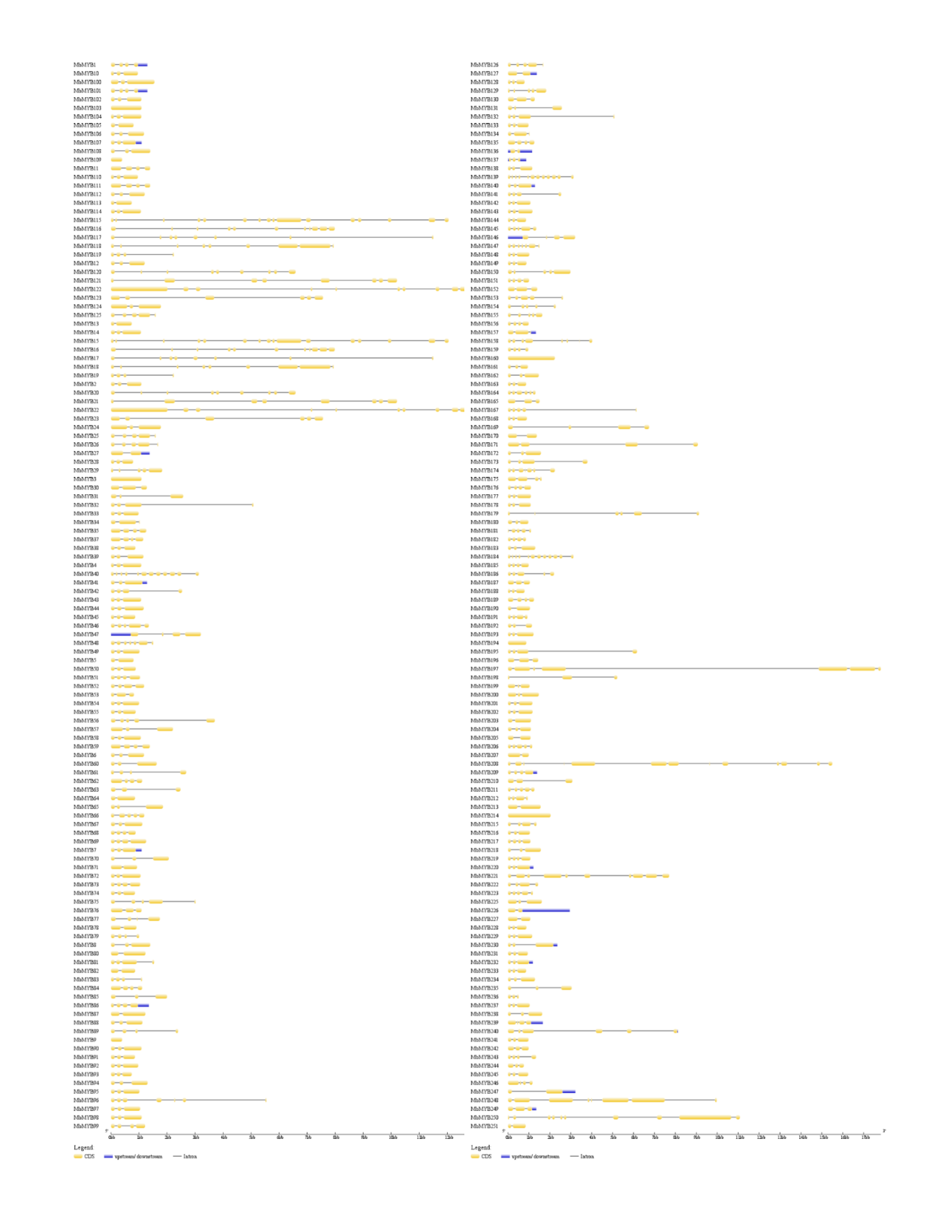

Supplement: Supplementary file 1 [file plants-09-00413-s001.zip › spm-plants-713656-revise1/S3.tif]

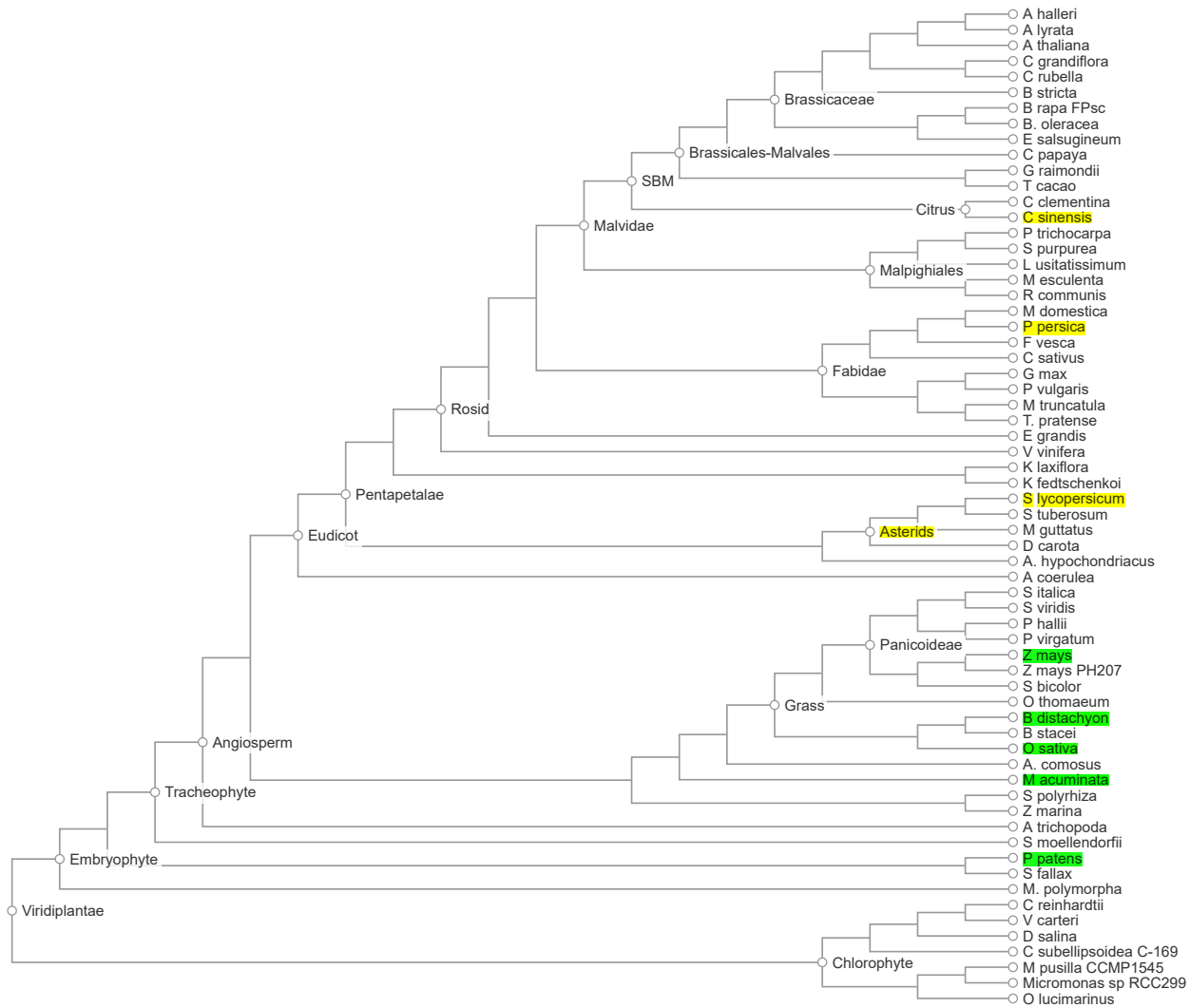

Supplement: Supplementary file 1 [file plants-09-00413-s001.zip › spm-plants-713656-revise1/Supp Figure S1.pdf]
